# Perception of Health Risks of Electronic Cigarette Use Among College Students: Examining the Roles of Sex, Field of Study, Vaping Device Type, and Their Associations

**DOI:** 10.1007/s10900-024-01393-y

**Published:** 2024-08-23

**Authors:** M. J. Ruzmyn Vilcassim, Samuel Stowe, Kristina Marie Zierold

**Affiliations:** Department of Environmental Health Sciences, UAB School of Public Health, 1665 University Boulevard, Birmingham, AL 35233 USA

**Keywords:** Electronic cigarettes, Vaping, College students, Perceptions, Comparative risks

## Abstract

Electronic cigarettes are marketed as a safer alternative to regular (combustible) cigarettes, based on the claim that there is no tobacco burning and fewer toxic chemicals in their vapor. However, recent evidence challenges the notion that e-cigarette aerosols are benign. Heating of compounds in e-liquids to high temperatures can lead to the release of toxic compounds in e-cigarette aerosols. However, users and the public may not be aware of these unique harms, impacting their perception of harm from using e-cigarettes. This research explored the perceptions of harm of e-cigarettes compared to regular cigarettes among 418 college students, aged 18–34, by employing a Qualtrics based smartphone survey. The findings revealed a vaping prevalence of 16.7% among our study population, indicating e-cigarette use among college aged young adults is at concerning levels. Perceptions of harm varied significantly by vaping status, sex, and field of study. Non-e-cigarette users and female students were less likely to perceive e-cigarettes as less harmful than regular cigarettes. Among e-cigarette users (vapers), male vapers and users of pod-type devices, such as JUUL and disposables, were more inclined to view e-cigarettes as less harmful. Among vapers, students in non-health-related fields were significantly more likely to perceive e-cigarettes as less harmful than regular cigarettes, underscoring the impact of educational background on health risk awareness. In conclusion, this study provides crucial insights into the varied perceptions of e-cigarettes among young adults. The results emphasize the need for targeted public health interventions and educational efforts to address this growing public health concern.

## Introduction

Electronic cigarettes (e-cigarettes) have had a significant impact on smoking habits and public health, transforming tobacco use behaviors and preferences among young adults, reflecting broader societal changes in attitudes toward electronic nicotine delivery systems (ENDS). While the prevalence of adult e-cigarette use in the United States has fluctuated from year to year, the overall prevalence grew substantially from 2.2% in 2012 to 4.5% in 2021 [1, 2]. E-cigarettes have been promoted as a safer alternative to regular combustible cigarette (hereinafter referred to as ‘regular cigarettes’) smoking and as a smoking cessation tool; however, this method of delivering nicotine via heating of an ‘e-liquid’, is now potentially an addictive behavior with its own unique harms [3]. Although the existing body of research generally supports the notion that e-cigarettes are less harmful than regular/combustible cigarettes, attributed primarily to the lack of combustion and relatively fewer toxic compounds in e-cigarettes aerosols compared to cigarette smoke [4, 5], this does not mean that e-cigarettes are void of risk. Numerous studies have found toxic compounds in e-cigarette vapor, including heavy metals, carbonyl compounds, polycyclic aromatic hydrocarbons (PAHs), and flavoring compounds that can potentially modify into more toxic compounds when heated [6–9]. Despite these findings, the rapid evolution of e-cigarette products and the introduction of new chemicals to e-liquids, necessitates ongoing research to ensure up-to-date assessments of their health impacts [10–12].

Public perception of e-cigarettes plays a crucial role in their usage trends and the regulatory landscape. The degree to which public perception aligns with scientific research can, at times, vary significantly, and be influenced by factors such as marketing strategies and target demographics [13]. Understanding people’s perceptions of e-cigarettes is particularly important as e-cigarettes are increasingly being used as an entry tobacco product by first-time tobacco users and young adults [14, 15]. This demographic shift is reflected in the substantial rise in e-cigarette use from 2012 to 2021 among young adults aged 18–24, among whom the prevalence increased from 2.4 to 11.0%, and also the increase in prevalence from 2.4 to 6.5% among adults aged 25–44 [1, 2]. This surge in e-cigarette use, particularly amongst young adults who have never used tobacco products before, suggests a need to further investigate young adults' perceptions of e-cigarettes to comprehend the factors driving their escalating use.

Some recent studies indicate a growing perception among adults that e-cigarettes are as harmful or more harmful than regular cigarettes. Malt et al. analyzed data from the Population Assessment of Tobacco and Health Study (PATHS) and found a decrease in the percentage of U.S. adults perceiving e-cigarettes as less harmful than regular combustible cigarettes from 41.1% in 2013 to 25.3% in 2016, while those seeing them as equally or more harmful increased from 53.7% to 72.7% during the same period [13]. Similarly, an analysis of data from the Health Information National Trends Survey (HINTS) from 2018–2020 and found that the percentage of U.S. adults who perceived e-cigarettes to be less harmful than cigarettes decreased from 17.6% in 2018 to 11.4% in 2020 while the perception that they are more harmful increased from 6.8% in 2018 to 28.3% in 2020 [16]. Additionally, some European and English studies reflect similar trends, showing most participants perceiving e-cigarettes as equally or more harmful compared to regular cigarettes [17, 18].

Contrarily, studies have also shown that some perceive e-cigarettes as being less harmful than regular cigarettes. Cooper et al. (2017) found that most college students (aged 18 – 29 years in the study) viewed e-cigarettes as minimally harmful compared to cigarettes. Several studies done in Hong Kong, Turkey, Australia, and the UK have revealed that young adults largely perceived e-cigarettes as less harmful and addictive than regular cigarettes [19–22].

Importantly, perceptions can vary among different user groups including between e-cigarette users, smokers, dual users, and non-users, as well as between daily and non-daily e-cigarette users [19, 23–25]. In the study by Cooper et al., students’ perceptions of e-cigarettes varied on user status, with exclusive e-cigarette users, exclusive cigarette smokers, and dual users all being significantly more likely to perceive e-cigarettes as posing little to no harm compared to non-users. Exclusive e-cigarette users and dual users were significantly more likely to perceive e-cigarettes as posing little to no harm than exclusive cigarette smokers [19]. A 2018 literature review by Romijnders et al. found that recent studies show both cigarette smokers and non-users are more likely to perceive e-cigarettes as equally or more harmful than regular cigarettes [23].

Further, recent events such as the E-cigarette or Vaping Product Use-Associated Lung Injury (EVALI) outbreak and the COVID-19 pandemic have potentially altered public perceptions of e-cigarettes [18, 26–29]. Several studies found that in 2019, when the EVALI outbreak occurred, and the years following the outbreak, participants’ perceived safety of e-cigarettes decreased while their perceived harm of e-cigarettes increased [18, 26, 28]. Similarly, the COVID-19 pandemic raised questions about the impact of e-cigarette use on respiratory health; during the pandemic e-cigarette users perceived themselves to be at greater risk for COVID-19 due to their e-cigarette use [27, 30–32]. These varying perceptions of e-cigarettes across different age groups and user statuses demonstrate a need for more nuanced research on the specific factors influencing these perceptions, particularly among young adults and current e-cigarette users.

This research aimed to understand perceptions of e-cigarettes compared to regular (combustible) cigarettes. We specifically focused on demographic and other characteristics associated with these perceptions, particularly among college students in the Deep South, where rates of cigarette smoking have been historically high. We evaluated what factors contribute to perceptions among e-cigarette users and non-users and relationships between these factors, thereby potentially identifying reasons for use and mitigation in the future. The insights gained from this research are expected to contribute to a better understanding of the dynamics behind the growing prevalence of e-cigarette use among young adults and inform future public health strategies and regulatory policies.

## Methods

The focus of this study was participants’ perception of e-cigarettes compared to regular cigarettes. To conduct a survey among college students aged 18 to 34 and attending universities in Alabama, we developed a Qualtrics^TM^ based questionnaire. Potential participants accessed the questionnaire via scanning a QR code in flyers that were posted across the University of Alabama at Birmingham’s campus, at key locations across the city of Birmingham, and shared via social media platforms. The survey was conducted to gather student’s opinions and behaviors concerning e-cigarettes and other tobacco products. Only participants who self-reported their age ≥ 18 were able to continue the online survey. Participant responses were recorded in Qualtrics under a unique identifier and no names or home addresses of participants were recorded. Data were collected from October 1, 2020, to October 20, 2023, in the Birmingham, AL metropolitan area. We have described the methods used including the questionnaire design, recruitment strategy, in detail, in a previous publication [33].

### Statistical Analysis

Data from participants were retrieved from Qualtrics into an Excel/.csv file, that recorded the data based on the assigned questionnaire numbers. Participants who were 18–34 years old and attended a university in the state of Alabama (UAB or other) were included in the analysis. Descriptive statistics were generated for the entire college student population, aged 18–34 years, and for exclusive e-cigarette users (vapers). Due to the limited number of participants who selected “I prefer not to answer” for their sex, (n = 2) we removed these respondents from the analysis. SAS 9.4 (Cary, NC) was used for all statistical analysis while figures were generated using R Studio Version 2024.04.2 + 764 running R version 4.3.2.

The primary outcome variable for this study was the perception of e-cigarettes compared to regular cigarettes and was categorized to three responses to the question “What is your perception of E-cigarettes/vaping compared to regular cigarettes” for which responses included: “They are less harmful than regular cigarettes” (1), “They are more harmful than regular cigarettes (2)” and “Not different from regular cigarettes (3)”. The reference for the logistic regression analyses was option (3): “Not different from regular cigarettes”. For some exposures of interest, we created either 2 or 3 level variables based on the number of participants in each category. These variables were coded as follows: Field of study or work: Non-health related (Engineering, Law, Business/Finance, Sciences, Arts, Skilled work, Other) = 0; Health related (Medicine, Nursing, Public Health) = 1; Grade in college: (Freshman + Sophomore) = 0, Junior = 1, and Senior = 2; Vaping device type used: Pod-type devices (Juuls + Disposable e-cigarettes) = 0 and All other type devices (Tanks/Mods/other rechargeable devices etc.) = 1.

To assess the differences in perception of e-cigarettes compared to regular cigarettes by the characteristics of the university e-cigarette users, Chi-square analysis or Fisher’s Test was used based on the sample size. Fisher’s test was used when cells had ≤ 5 participants. To evaluate the magnitude of the perceptions of e-cigarettes compared to regular cigarettes, the characteristics of the participants (sex, race, grade in university, field of study, place of residence), and vaping device used, logistic regression was used to calculate Odds Ratios (OR) and 95% Confidence Intervals. Additional covariates such as number of friends using e-cigarettes and age starting vaping were investigated as potential confounders and were not found significant in simple models; therefore, no adjustments were made in the final models.

## Results

### Descriptive Statistics

There was a total of 418 students who participated in the survey. Of those, 70 reported current exclusive e-cigarette use, demonstrating an estimated vaping prevalence of 16.7% among our 18–34-year-old college student population. Table 1 shows the demographics of our study. Our total sample had a higher percentage of female students (74%) and most students self-reported as White. Despite the higher percentage of female students in the main population, e-cigarette usage was almost evenly split among male and female students (approximately 51% and 49%, respectively)., When assessing e-cigarette use by year in college students that reported being juniors (33%) had the highest prevalence of use, followed by seniors (24%) When the main field of study was considered, students in the field of Sciences exhibited the highest rate of e-cigarette use. Amongst vapers, disposable e-cigarettes were the most used vaping device type followed by Tanks/Mod type devices and JUUL (45%, 20% and 16%, respectively). Other demographic statistics of our study population have been reported in a previous publication [33].

**Table 1:** Demographics characteristics of college students, 18–34 years old (N = 418)

Demographics	College students in total sample, N = 418 N, (%)	Among all college students E-cigarette users only, N = 70 N, (%)
Sex		
Female	309 (73.9%)	36 (51.4%)
Male	109 (26.1%)	34 (48.6%)
Self-reported race		
White	246 (60.0%)	53 (79.1%)
Black	77 (18.8%)	4 (6.0%)
Middle eastern	11 (2.7%)	2 (3.0%)
South Asian	33 (8.0%)	3 (4.5%)
East Asian	23 (5.6%)	2 (3.0%)
Other	20 (4.9%)	3 (4.5%)
Year in college		
Freshman	66 (15.8%)	6(8.6%)
Sophomore	86 (20.6%)	14 (20.0%)
Junior	117 (28.0%)	23 (32.9%)
Senior	94 (22.5%)	17 (24.3%)
Graduate student	42 (10.0%)	7 (10.0%)
Other	13 (3.1%)	3 (4.3%)
Field of study		
Medicine	71 (17.0%)	6 (8.6%)
Nursing	28 (6.7%)	4 (5.7%)
Engineering	25 (6.0%)	5 (7.1%)
Law (pre)	5 (1.2%)	3 (4.3%)
Business	32 (7.7%)	9 (12.9%)
Sciences	91 (21.8%)	22 (31.4%)
Arts	20 (4.8%)	4 (5.7%)
Public health	119 (28.5%)	14 (20.0%)
Other	27 (6.5%)	3 (4.3%)

Percentages may not add to 100%, due to missing values

### Perception of Harm of e-Cigarettes Compared to Regular Cigarettes Among All College Students

**Fig. 1:**
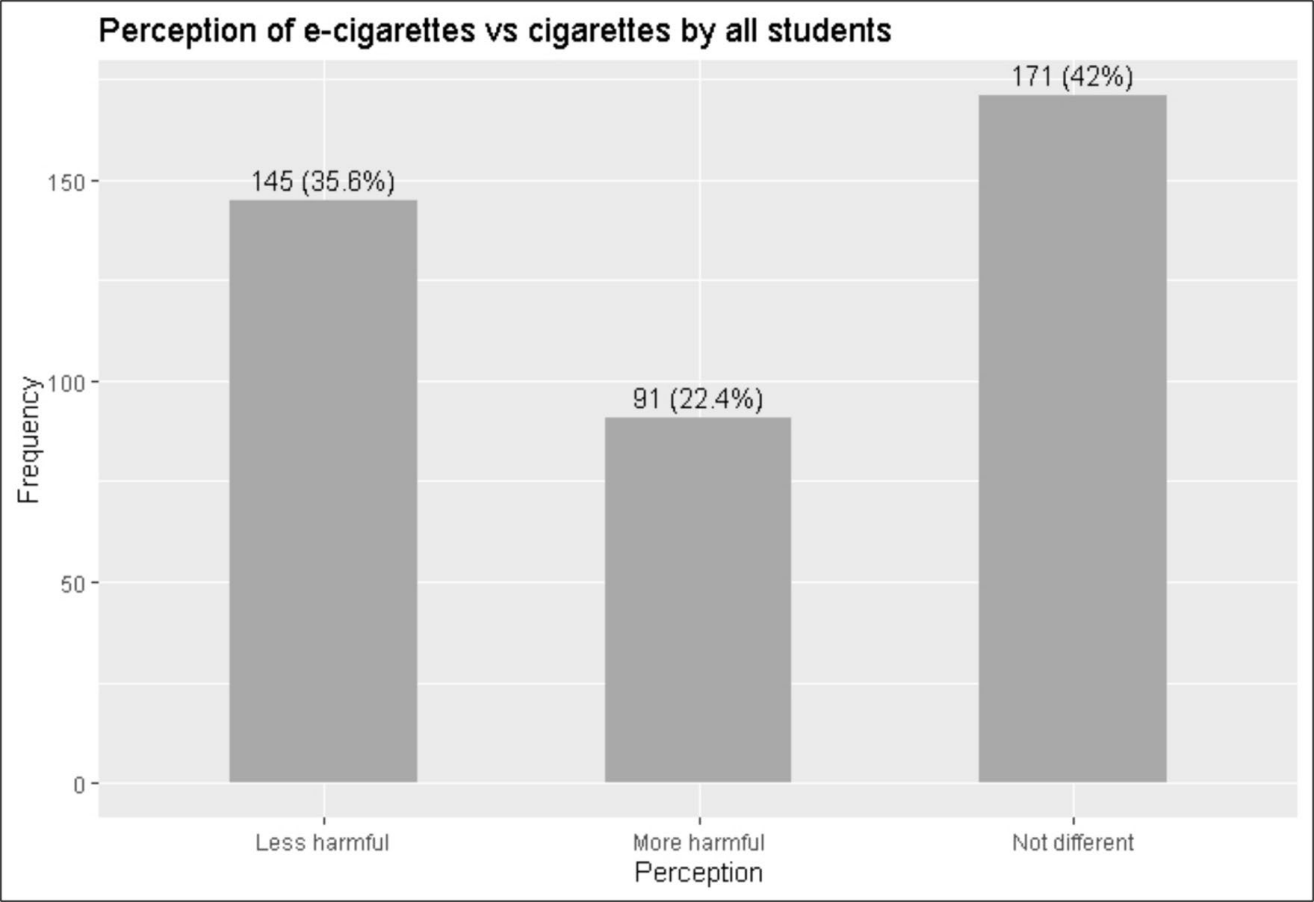
Perception of e-cigarettes compared to regular cigarettes among all college students in our study sample (n = 418) (Eleven participants did not respond to the question on perception and therefore, the total does not add up to 418 in the graph)

Overall, among all college students aged 18–34 years old, who responded to our survey (N = 418), 145 individuals perceived e-cigarettes to be less harmful than regular cigarettes, 91 individuals perceived e-cigarettes to be more harmful than regular cigarettes, and 171 individuals perceived no difference between e-cigarettes and regular cigarettes (Fig. 1). Perceptions based on user characteristics were further analyzed and described below.

### Perceptions Based on Vaping Status

Among the entire population in our study (N = 418), non-vapers were significantly less likely to believe that e-cigarettes are less harmful than regular cigarettes (OR = 0.253, 95% CI 0.130–0.493) compared to vapers.

Although not statistically significant, our analyses showed that among our population of university students who were non-vapers, they were two times more likely to believe that e-cigarettes are more harmful than regular cigarettes (OR = 2.33, 95% CI 0.647–8.29).

### Perceptions Based on Student Sex

Female students were significantly less likely (OR = 0.434, 95% CI 0.259–0.729) to believe that e-cigarettes are less harmful than regular cigarettes compared to male students. Additionally, female students were also three times more likely (OR = 2.95, 95% CI 1.25–6.98) to believe that e-cigarettes are more harmful than regular cigarettes, compared to male students.

### Perception of Harm of e-Cigarettes Compared to Regular Cigarettes Among Those Who Use e-Cigarettes (Among Exclusive Vapers)

**Fig. 2:**
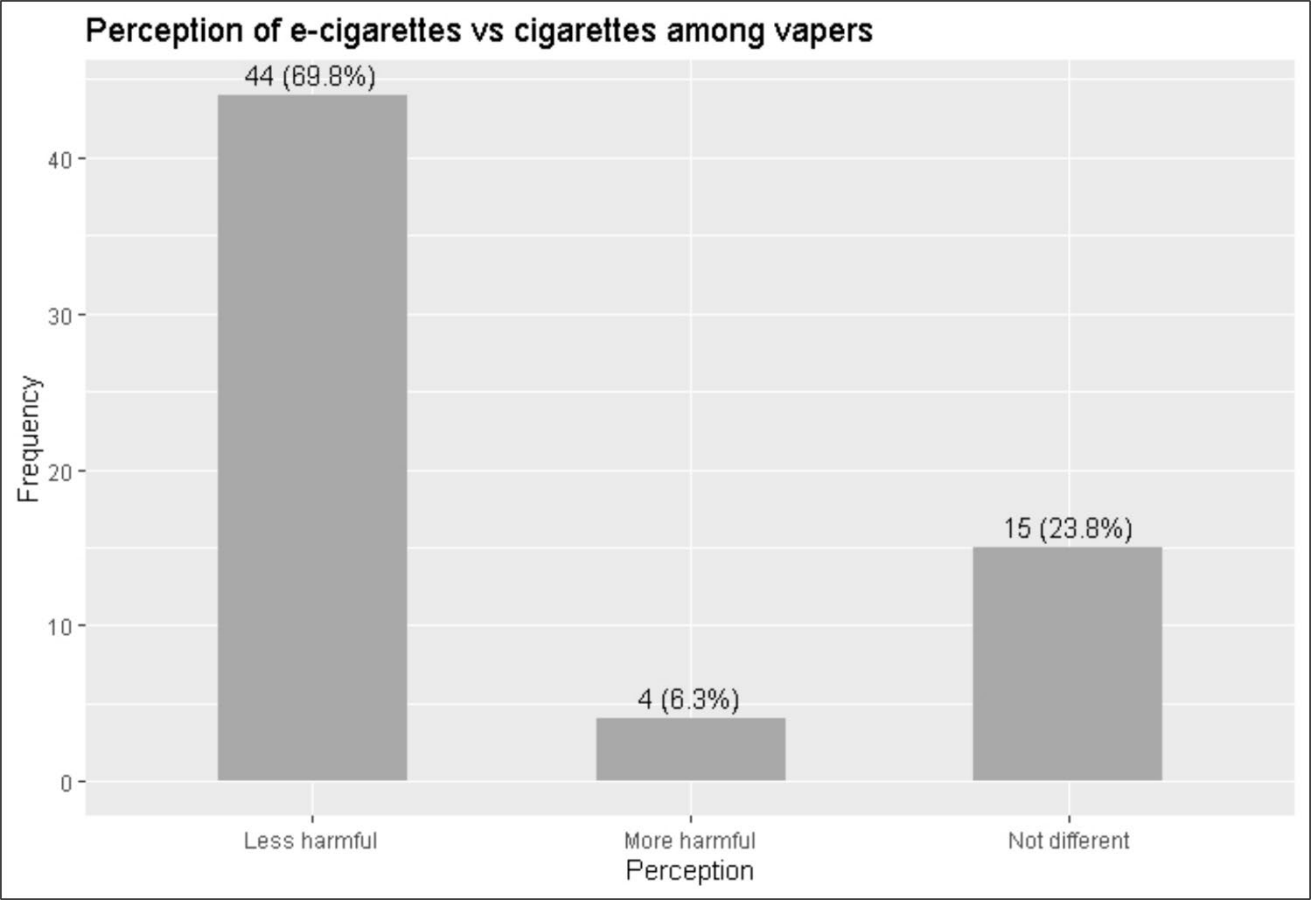
Perception of harm caused by e-cigarettes compared to regular cigarettes among those who use e-cigarettes (among vapers) (n = 70) (Seven vapers did not respond to the question on perception, and therefore, the total number of responses does not add up to 70 in the graph)

Among vapers, 44 individuals perceived e-cigarettes to be less harmful than regular cigarettes, 4 individuals perceived e-cigarettes to be more harmful than regular cigarettes, and 15 individuals perceived no difference in harm between e-cigarettes and regular cigarettes (Fig. 2). Perceptions of harm were further analyzed based on user sex, field of study, and preferred/primary vaping device, and are described below.

### Perceptions Based on User Sex

Among vapers only, males were approximately 4.7 (OR = 4.685, 95% CI: 1.13 – 19.34) times more likely to perceive e-cigarettes as less harmful than regular cigarettes, compared to females.

### Perceptions Based on Field of Study/Work

Among vapers, students in non-health fields (Engineering, Law, Business/Finance, Sciences, Arts, Skilled work, Other) were significantly more likely (OR = 1.98, 95% CI 1.26–3.12) to believe that e-cigarettes are less harmful than regular cigarettes, compared to students in health-related fields (Medicine, Nursing, Public Health).

### Perceptions Based on e-Cigarette Device Type Used

Among vapers, students who primarily used pod type devices were approximately three times more likely (OR = 2.93, 95% CI 1.34–6.43) to believe that e-cigarettes are less harmful than regular cigarettes, as compared to those who used other types of e-cigarettes (Tanks, Mods, and other rechargeable type e-cigarettes).

## Discussion

The findings from this research contribute to the efforts to understand and mitigate factors contributing to high rates of e-cigarette use among young adults, attending college. Results showed that vaping prevalence among our study population is 16.7%, which confirms that vaping rates among young adults remain at a high level, as also demonstrated in other similar studies. However, the rate reported in our study is higher than some previously reported rates in past years [1, 2], indicating that vaping rates among college aged young adults may be on the rise, particularly in the Deep South.

Overall, among all college students in our research demonstrates that the perception of the potential harm of e-cigarettes compared to regular cigarettes can vary based on several factors, including sex of users, vaping status of users, and field of study and/or work. One key observation was that non-e-cigarette users and female students were less likely to perceive e-cigarettes as less harmful than regular cigarettes. This could be attributed to a few factors. Non-users may rely more heavily on public health messages and media reports, which often emphasize the risks associated with vaping. The numerous health warnings, and news about the health impacts of e-cigarettes in recent events, such as the EVALI outbreak and the COVID-19 pandemic, likely reinforces the perception of harm. Female students, on the other hand, may be more risk-averse, potentially leading to a higher perception of harm. Males also tend to engage in riskier behaviors [34], and therefore, their use of tobacco products may be increased, as they may not pay as much attention to the public health messages regarding vaping.

Among vapers, the type of e-cigarettes used by the students, their sex, and field of study/work played a key role in their perception of harm caused by e-cigarettes. Male vapers were more likely to perceive e-cigarettes as less harmful than regular cigarettes compared to female vapers. Again, this could be due to differing risk perceptions or information sources between sexes [34]. Additionally, Pod type e-cigarette users, which included JUUL and disposable type e-cigarette users, were more likely to believe that e-cigarettes are less harmful than regular cigarettes. Our previous research demonstrated that there was a significant difference between sexes in the vaping device types used, where female students preferred disposable and pod type devices and male students preferred Tank/Mod type devices [33]. These preferences in device type may have been influenced by perception of harm or vice versa. Pod type devices typically have a smaller volume of e-liquid and are discarded after a single or few times use compared to larger Tank/Mod type devices that have a larger volume of e-liquid and discarded after a single or few users compared to larger Tank/Mod type devices with larger volumes. It is also important to note that in recent times, smaller pod-type devices had seen a rapid rise in sales [35] and marketed aggressively by e-cigarette companies as a safer alternative, targeting youth. It is highly possible that these marketing strategies and various claims by e-cigarette manufacturers impact perceptions, at times overshadowing scientific evidence.

The field of study also played a crucial role in perceptions. Interestingly, students in non-health fields were two times more likely to perceive e-cigarettes to be less harmful than regular cigarettes. This difference could stem from a lack of exposure to health-related information and education about the risks associated with e-cigarettes among students in non-health fields. Students in health-related fields might be more knowledgeable about the latest research and the potential health impacts of vaping, leading to a more cautious perception. However, recent research has shown that, toxicologically, e-cigarettes contain fewer harmful chemicals compared to regular cigarettes [4, 5]. Whether this information is known to students in non-health and health related fields is unclear and should be further explored.

This study has some limitations that should be considered when interpreting the findings. First, the self-reported nature of the data may have introduced the possibility of response bias. Participants may have provided socially desirable answers or may not accurately recall their behaviors and perceptions. Second, the study was conducted in a metropolitan area in the Deep South, which may limit the generalizability of the findings to other regions or populations. The cultural, social, and economic factors unique to this area, which have historically had high levels of smoking, might influence vaping behaviors and perceptions differently than in other parts of the country or world. Third, the sample size, although sufficient for the primary analyses, becomes limited when divided into subcategories. This limitation resulted in wide confidence intervals for some associations, indicating less precision in these estimates. Larger sample sizes would allow for more robust analyses and conclusions. Fourth, the recruitment locations, being university-centric, might have influenced the population characteristics. The study population primarily consisted of university students, who may not represent the broader population of young adults. University students might have different access to information, educational backgrounds, and social environments compared to their non-student peers, which can influence their perceptions and use of e-cigarettes. Fifth, recent events and media portrayals of vaping, and events such as the EVALI outbreak and the COVID-19 pandemic, could have significantly impacted participants' perceptions. Finally, the categorization of e-cigarette devices into pod-type and tank/mod-type might oversimplify the diversity of devices and usage patterns. The vaping experience can vary widely based on device type, e-liquid composition, and user behavior, all of which can influence harm perceptions. These devices are also fast evolving, creating a moving target for users as well as researchers, complicating research efforts on understanding risks of use. Longitudinal studies would be valuable in understanding how perceptions evolve, particularly in response to new research findings, regulatory changes, and public health campaigns. Tracking changes in perceptions over time would help in developing more effective intervention strategies to address misconceptions about e-cigarettes.

## Conclusions

In summary, our research highlights the complex and varied perceptions of harm from e-cigarettes compared to regular cigarettes among young adult college students, which is the demographic age group that has the highest prevalence of e-cigarette use worldwide. Our findings reveal that non-e-cigarette users and female students are less likely to believe that e-cigarettes as less harmful than regular cigarettes, possibly due to heightened awareness of health risks and reliance on public health information. Conversely, male vapers and users of pod-type devices are more likely to perceive e-cigarettes as less harmful than regular cigarettes, possibly influenced by marketing strategies, device characteristics, and risky behavior of males. The field of study also played a significant role, with students in non-health fields more likely to underestimate the risks associated with e-cigarette use. These results underscore the need for targeted public health interventions that provide accurate information about the potential risks of e-cigarettes, especially among young adults and university students. Future research should continue to explore the underlying factors influencing these perceptions to develop more effective strategies for reducing e-cigarette use in this population.
